# Protoplasts: From Isolation to CRISPR/Cas Genome Editing Application

**DOI:** 10.3389/fgeed.2021.717017

**Published:** 2021-08-11

**Authors:** Jin-Jun Yue, Jin-Ling Yuan, Fu-Hui Wu, Yu-Hsuan Yuan, Qiao-Wei Cheng, Chen-Tran Hsu, Choun-Sea Lin

**Affiliations:** ^1^ Research Institute of Subtropical Forestry, Chinese Academy of Forestry, Hangzhou, China; ^2^ Agricultural Biotechnology Research Center, Academia Sinica, Taipei, Taiwan

**Keywords:** RNP, transient transfection, DNA-free, CRISPR/cas (clustered regularly interspaced short palindromic repeats), protoplasts

## Abstract

In the clustered regulatory interspaced short palindromic repeats (CRISPR)/CRISPR associated protein (Cas) system, protoplasts are not only useful for rapidly validating the mutagenesis efficiency of various RNA-guided endonucleases, promoters, sgRNA designs, or Cas proteins, but can also be a platform for DNA-free gene editing. To date, the latter approach has been applied to numerous crops, particularly those with complex genomes, a long juvenile period, a tendency for heterosis, and/or self-incompatibility. Protoplast regeneration is thus a key step in DNA-free gene editing. In this report, we review the history and some future prospects for protoplast technology, including protoplast transfection, transformation, fusion, regeneration, and current protoplast applications in CRISPR/Cas-based breeding.

## Introduction

Many genes and single-nucleotide polymorphisms (SNPs) related to important phenotypes have been identified by an array of bioinformatic tools utilizing the rich and diverse genome resources currently available (Varshney et al., 2009). These regions can then be experimentally altered through targeted mutagenesis, single-base-pair substitution, or DNA insertion using clustered regulatory interspaced short palindromic repeats (CRISPR)/CRISPR associated protein (Cas)-mediated gene editing methods. CRISPR/Cas is very convenient, requiring only a Cas protein and a single guide RNA (sgRNA) designed to target the sequence of interest. For stable transformation, these two reagents are delivered to plant cells by gene transformation using *Agrobacterium tumefaciens* (Agrobacterium) or biolistics. DNA encoding the Cas protein and the sgRNA genes is inserted into the plant genome and expressed (Zhu et al., 2020). Selection markers can be used to screen for transformed plants, making it necessary to backcross the selected progeny with the original parental line to segregate out/eliminate foreign DNA. This will be very time consuming and expensive, especially for crops with polyploidy, long juvenile periods, a tendency for heterosis, or self-incompatibility, and back-crossing itself can cause divergent phenotypes in offspring, including a relatively long flowering time.

More recently, scientific developments have allowed gene modification using transient expression of the Cas protein and sgRNA, with no need for insertion into the chromosome. Reagents can be delivered to intact somatic plant cells either as DNA or ribonucleoprotein (RNP) using biolistic delivery (Liang et al., 2018), nanotubes (Demirer et al., 2019), virus transfection (Ellison et al., 2020), or *Agrobacterium* infiltration (Chen et al., 2018) without DNA insertion. These edited cells are then grown into edited, regenerated plants by tissue culture or by controlling the expression of growth regulation genes (Maher et al., 2020). One additional approach is to deliver CRISPR reagents directly into plant zygotes using polyethylene glycol–calcium (PEG–Ca^2+^; Toda et al., 2019); this yields high editing rates, but plant zygotes are small and difficult to manipulate.

Protoplasts, plant cells without cell walls, have been widely used in plant science research and crop breeding, and protoplast transfection (*via* PEG–Ca^2+^ and electrophoresis) can achieve high efficiency without a selection marker (Marx, 2016). The genome editing reagents (DNA, RNA, RNP) can be delivered into protoplasts via transfection; therefore, protoplast transfection is commonly used in model organisms and crops to test the efficiency of gRNA design, and Cas protein activity (Lin et al., 2018; Lin et al., 2020; Sretenovic et al., 2021). Furthermore, these edited protoplasts can be regenerated into plants. However, only a few studies on this method have been published, and most have involved dicotyledonous species (Woo et al., 2015, Andersson et al., 2017; Lin et al., 2018; Hsu et al., 2019; Park et al., 2020; Yu et al., 2020; De Bruyn et al., 2020; Hsu et al., 2021a; Hsu et al., 2021b). Despite protoplast isolation, regeneration, transfection, and transformation protocols having been established for many years, lack of protoplast regeneration systems for target crops remains major challenge for widespread utilization of protoplast transfection for DNA-free genome editing. In this mini review, we outline both historical and current results and demonstrate that protoplast regeneration technologies have developed to the point that CRISPR/Cas-based modification of protoplasts is a viable gene editing platform.

## Protoplast Isolation

In 1892, Klercher was the first to isolate protoplasts (reviewed by Cocking, 1972; Davey et al., 2005) by using a mechanical method to remove plant cell walls. In this approach, an onion bulb (*Allium cepa*) is sliced and placed in a plasmolysing solution to pull the membranes of epidermal cells away from their walls. Tissues are then placed on a slide and cut with a blade. Many cells that have one end of their cell wall cut off will still contain intact, plasmolysed cells. Protoplasts can then be isolated by removing these intact plasmolysed cells from the remaining cell wall (Chambers and Höfler, 1931). However, this method is only feasible for storage tissues such as bulbs; meristematic cells require more extensive plasmolysis and only yield a small number of protoplasts (Cocking 1972; Davey et al., 2005).

In 1919, Giaja demonstrated that yeast protoplasts could be isolated by using snail gastric juice to digest their cell walls (reviewed by Cocking, 1972). This enzymatic method was first applied to bacteria, algae, and fungi. Cocking (1960) expanded the method to multicellular plants when he used purified fungal cellulase to create protoplasts from tomato root tips, which contain meristematic cells. There were many advantages of this enzymatic method over the prior, mechanical method; more protoplasts could be obtained, and the tissue was subjected to less mechanical damage and osmotic shrinking (Ruesink, 1971; Cocking, 1972).

At present, the primary method for protoplast isolation is based on Cocking’s enzymatic method, in which cells are first plasmolysed by mannitol and then digested by macerozyme and cellulase. Leaves are by far the most convenient material for protoplast isolation. The leaves are cut into strips, and the lower epidermis is braced or peeled off to allow the enzymes to enter the inter-mesophyll space to enhance cell wall digestion. We developed a simple protocol, the Tape-Arabidopsis Sandwich, in which the lower epidermal layer of an *Arabidopsis thaliana* (Arabidopsis) leaf is removed with regular office tape to expose mesophyll cells to cell-wall-digesting enzymes (Wu et al., 2009). This innovation allows protoplasts to be obtained with less physical damage and can also make protoplast isolation more convenient. We also developed a multi-blade tool for cutting leaves into thin strips to improve rice protoplast isolation (Lin et al., 2018).

Other plant organs can also be used as materials for protoplast isolation, such as roots, stems, leaves, flowers, pollen, fruit pulp, and embryos. Protoplasts derived from developed organs retain the properties of the original organs and can be more suitable than the protoplasts derived from other organs for use in physiological and biochemical experiments (Lin et al., 2014). In addition to plant materials, suspension culture cells like tobacco BY2 (Nagata et al., 1992) and tomato MicroTom (Lin et al., 2018) cells can be used to improve the consistency of protoplast isolation or to make it more convenient. Cell-culture-derived protoplasts are commonly used to overcome plant growth limitations specific to the experiments that require a large number of protoplasts (Lee et al., 2008).

## Protoplast Regeneration

**FIGURE 1:**
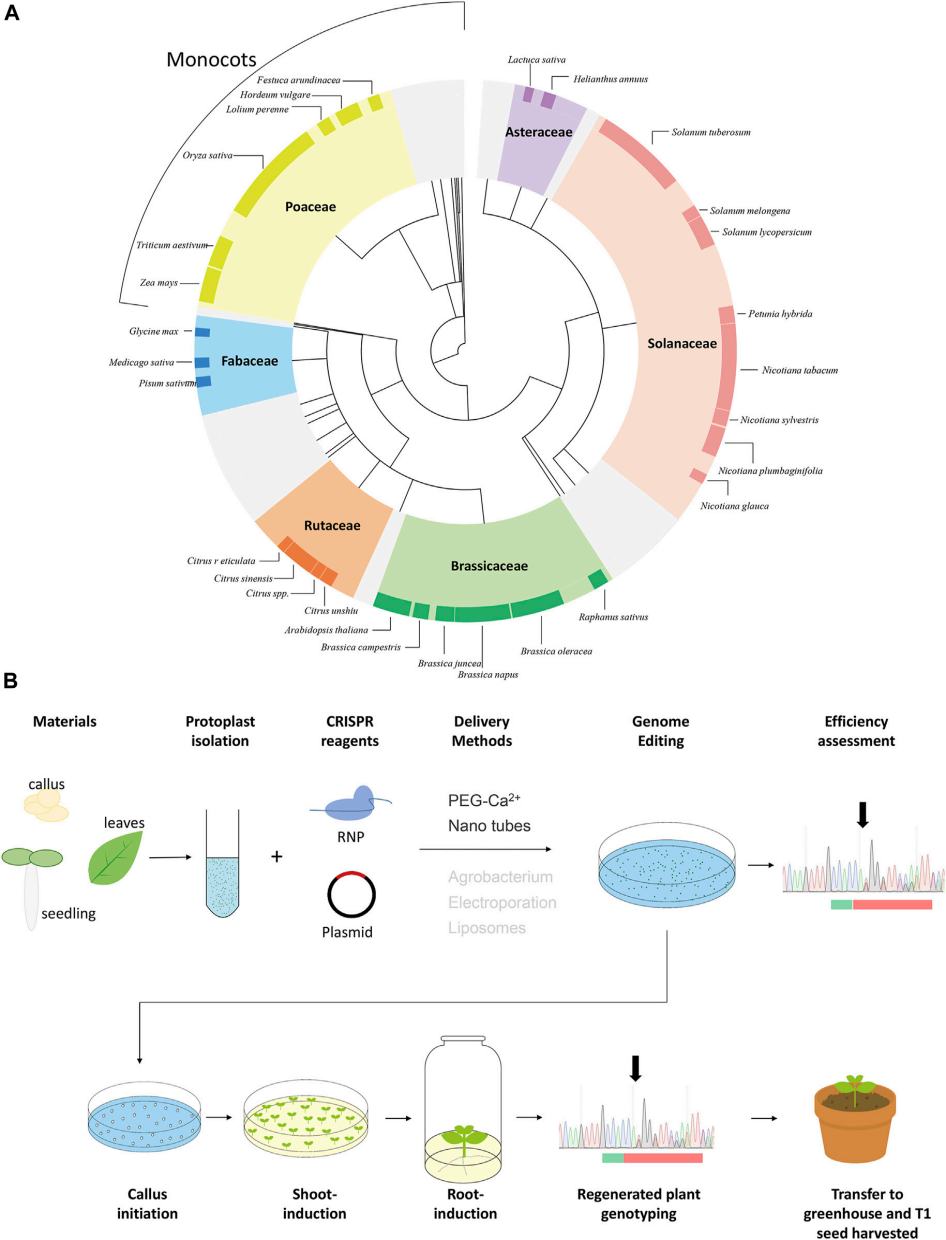
Protoplast regeneration and CRISPR genome editing. **(A)** Protoplast regeneration-related articles according to taxonomy. These 779 protoplast regeneration articles were identified from Google scholar and NCBI (Supplementary Table S1). The taxonomy of the plant species follows the Angiosperm Phylogeny Group IVsystem and NCBI taxonomy. Major familes are color coded as follows: purple; Asteraceae, light orange; Solanaceae, green; Brassicaceae, orange; Rutaceae, blue; Fabaceae, yellow-green; Poaceae. The names of the species reported in more than five articles are shown. The rest of the species are shown in grey. The information used to create this diagram is shown in Supplementary Table S1. **(B)** Schematic of tobacco protoplast regeneration. Delivery methods stated in grey are the methods used for protoplast transformation that can theoretically be applied in genome editing. The multiple peaks (black arrows in “Efficiency assessment” and “Progeny genotyping”) in the upstream of PAM (green boxes) are indicated. tThe target site (red boxes) is editied by CRISPR reagent.

Protoplasts isolated from totipotent meristematic cells were first used for plant regeneration in the early 1970s (Takebe et al., 1971). To understand research trends within the protoplast regeneration field, we analyzed 779 protoplast-regeneration-related articles (Figure 1A; Supplementary Table S1). Protoplasts are most frequently made from plants in the Solanaceae, Poaceae, and Brassicaceae families. This is because protoplast regeneration in these families tends to be easier, and also because many economically important crops belong to these families (i.e., rice is from the Poaceae, potato is from the Solanaceae, and *Brassica oleracea* is from the Brassicaceae). The protocol details, including explant (tissues from donor plants) source, culture incubation system, protoplast density, basal medium, growth regulators, and supplements, are optimized for each species and sometimes for each variety. For example, most protocols use juvenile organs as explants, such as seedlings for the Brassicaceae (Gerszberg et al., 2015) and cell suspensions for monocots (Abdullah et al., 1986; Supplementary Table S1). However, in tobacco and other Solanaceous species, a regeneration protocol was established using mature leaves because, in contrast to other species, this is easier and is effective for the Solanaceae. Thus, different species, and even different varieties of the same crop, require the establishment of their own protoplast regeneration protocols.

According to a review by Roest and Gillissen (1989), the number of articles related to protoplast regeneration peaked in 1989. More recently, the numbers have decreased significantly. This is because the development of species-specific protocols is a technical barrier that prevents the widespread use of protoplasts (Eeckhaut et al., 2013).

## Protoplast Fusion

Two protoplasts (Kiister, 1909) can be fused into a single cell (see the review by Constabel, 1976) even if they are from different species, and the efficiency of such fusion can be increased by using Na_2_CO_3_ (Power et al., 1970), PEG (Kao et al., 1974), or electrofusion (Zimmermann and Scheurich, 1981). These fused protoplasts can be regenerated into plants that are somatic hybrids of the two original species (e.g., *Nicotiana glauca* and *N. langsdorffii*, Carlson et al., 1972). This strategy became a solution for crop breeders in cases where sexual incompatibility was a barrier or as a means to incorporate traits from wild species into related crops without the need for sexual reproduction (Louzada et al., 1993). Variations on this method have been used to introduce a variety of desired traits into crops, including stress resistance (Hennig et al., 2015), pathogen resistance (Kumari et al., 2020), seedlessness (Wu et al., 2005), male sterility (Bruznican et al., 2021), and increased photosynthetic efficiency (Takahata and Takeda, 1990). This can be performed to create a symmetric cell fusion, in which the complete nuclear genomes of the two species are combined (Narasimhulu et al., 1992; Laiq et al., 1994; Ling and Iwamasa, 1994; Desprez et al., 1995; Kirti et al., 1995); an asymmetric cell fusion, in which DNA fragments or partial chromosomes from one species are introduced into the other (Zhou and Xia 2005; Sigeno et al., 2009); or a cybrid, in which chloroplast or mitochondrial genomes from one species are introduced into cells of another (Kochevenko et al., 2000; Guo et al., 2004). Not only intra-genus, one especially interesting case was a monocot-dicot (*Triticum aestivum* and Arabidopsis) protoplast fusion in which regenerated calli and green plants resembling that of wheat were obtained (Deng et al., 2007). However, protoplast fusion has become less common in recent years, primarily because plant molecular genetic research over the past few decades has identified many key genes controlling important traits and thus enabled the use of more targeted approaches.

## Protoplast Transformation

For stable transformation of a protoplast, foreign DNA must be integrated into its genome. This can be achieved by using the crown-gall-inducing bacterium *Agrobacterium* to transfect plant tissues with its Ti plasmid (Marton et al., 1979; Wullems et al., 1981a; Wullems et al., 1981b). Plants were regenerated, and analysis of their progeny indicated that the tumor markers were inherited through meiosis (Wullems et al., 1981a; Wullems et al., 1981b). Davey et al. (1980) obtained transformants using purified Ti plasmid. To increase the transformation efficiency, Krens et al. (1982) used a PEG-mediated method for protoplast transformation. Antibiotic resistance genes can be cloned into the Ti plasmid and used as selectable markers for the transgenes of interest (Paszkowski et al., 1984). Shillito et al. (1985) investigated and optimized the parameters for both a PEG-based and an electroporation protocol. Transformation efficiency was increased by 1,000-fold, to 2%, without selection. However, the stable transformation and regeneration of protoplasts has proven to be more difficult to establish than other methods. Currently, the most popular methods for stable plant transformation are *Agrobacterium*- and biolistics-mediated transformation by somatic embryogenesis and organogenesis.

## Protoplast Transfection

Since protoplasts lack the barrier of a cell wall, they can easily take up foreign DNA or protein, making them excellent material for transient expression or stable transformation (Lazzeri et al., 1991; Sheen 2001; Yoo et al., 2007). Transient transfection can also allow foreign genes to be expressed in protoplasts for a short period of time to observe phenotypes such as *in vivo* gene expression, protein localization, DNA-protein interaction, or protein-protein interaction. There are approximately 2,000 articles referring to the use of the Transient Expression in Arabidopsis Mesophyll Protoplast (TEAMP) system for basic research (Yoo et al., 2007), and we have developed and optimized isolation and transfection protocols for important crops (Lin et al., 2018). Both protocols are applicable for CRISPR studies.

## CRISPR and Protoplasts

Because of the convenience of protoplast transfection, it has been used to assess the mutagenesis efficiency of the CRISPR/Cas system. The Cas nuclease most commonly used is Cas9 (PAM requirement: NGG), but Cas12a (PAM requirement: TTTN) has been employed in rice, tobacco, and soybean protoplasts (Kim et al., 2017; Tang et al., 2017; Hsu et al., 2019) to increase the DNA regions that can be edited. Cas13 can also be used for RNA gene editing and has been examined in rice protoplasts (Abudayyeh et al., 2017). Transient expression has been used to assess and optimize CRISPR protocols, including validation of Cas codon-optimization or modification, sgRNA, identification of the best promoter and analysis of different vector designs (Li et al., 2013; Shan et al., 2013; Lowder et al., 2015; Čermák et al., 2017; Nadakuduti and Enciso-Rodriguez 2021).

When validating CRISPR efficiency, more than 100,000 protoplasts are typically used in each transfection experiment. This large pool of protoplasts contains a mixture of edited and unedited DNA, which complicates the evaluation of editing efficiency (Lin et al., 2018). Editing efficiency can also be assessed using next-generation sequencing. This method, although accurate, is expensive and time consuming. As an alternative, we recently established a convenient and reliable protocol to quantify the efficiency of a CRISPR procedure that uses only a single protoplast (Lin et al., 2018), in which a single cell is picked up by a lab pipette and subjected to two rounds of PCR to obtain enough DNA for genotyping. This single-protoplast analysis improves the accuracy with which editing efficiency can be evaluated.

While there are many articles reporting crop CRISPR/Cas stable transformation platforms, these efforts are complicated by the fact that many commercial crop varieties are polyploid, heterozygous, or asexually propagated. Because of the back-crossing required to eliminate the CRISPR transgene, the development of CRISPR-mediated transgenic crops is limited by the complex genome, long juvenile period, and/or self-incompatibility of many commercial varieties. In these crop varieties, CRISPR-mediated, DNA-free genome editing in protoplasts followed by regeneration into whole plants would be the most feasible way to directly apply gene editing technologies to improve traits and increase commercial value. This method has already been experimentally proven in protoplasts including potato (Andersson et al., 2017; Andersson et al., 2018; Tuncel et al., 2019; González et al., 2020; Zhao et al., 2021; Nicolia et al., 2021), *N. tabacum* (Lin et al., 2018; Hsu et al., 2019), *N. benthamiana* (Hsu et al., 2021a,b), *Brassica oleracea* (Park et al., 2020; Hsu C. T. et al., 2021), lettuce (Woo et al., 2015), petunia (Yu et al., 2020), and witloof (De Bruyn et al., 2020). The main steps of gene editing using protoplast regeneration are illustrated in Figure 1B.

There are two major methods to regenerate plants from CRISPR-mediated edited protoplasts. Protoplasts can be transfected with plasmid DNA, or they can be transfected with preassembled RNP. However, when protoplasts are transiently transfected with DNA, a substantial proportion of the regenerated plants contain unintended inserts from the CRISPR plasmid (Andersson et al., 2017; Hsu et al., 2019). The use of RNP removes the risk of plasmid DNA insertions into the plant genome because there is no foreign DNA during the transfection (Andersson et al., 2018). These results illustrate the potential and feasibility of using protoplasts for CRISPR-meditated gene editing, especially for crops that have a long juvenile phase, are heterozygous, or are asexually propagated. Using protoplasts for CRISPR modification means that gene editing products occur directly in the T_0_ generation without foreign CRISPR DNA and without the need for hybridization, introgression, or back-crossing of the progeny. Recently, *Agrobacterium*-mediated expression without the use of antibiotic selection has also been adapted into a transgene-free protocol, which could be a very promising track for future development of this technology (Chen et al., 2018).

Most *Agrobacterium*-mediated transformation protocols are performed on tissue culture platforms, and, in dicots, many edited transformants are chimeric (33.3–81.8%; Shimatani et al., 2017). If edited alleles are not present in the reproductive organs, the changes cannot be passed on to the progeny. In contrast, protoplasts are single cells that are edited before the first cell division occurs. Regenerated plants are derived from a single edited protoplast, meaning all cells have the same genomic background and ensuring edited alleles are transmitted to the next generation. In our previous study, non-chimeric regenerates were derived from protoplasts that had been edited using the Cas proteins Cas9, Cas12a, and Target-AID, and the resulting genotypes were inherited in a Mendelian manner (Hsu et al., 2019). Another group has achieved this feat for lettuce (Woo et al., 2015).

## Discussion

In most crop species, protoplast regeneration is still a technical barrier. Meanwhile, protoplast-regenerated plants sometimes show abnormal, stunted growth, probably due to somaclonal variation. Whole-genome sequencing results indicate that there is widespread genome instability in protoplast-regenerated potatoes (Fossi et al., 2019), which has increased concern about this technology. However, other tissue culture technologies, including multiple shoot proliferation (Lin and Chang 1998) and somatic embryogenesis (Lin et al., 2004), can also cause mutations. Also, polyploid plants can arise as a result of other tissue culture technologies (Chung et al., 2017). In Agrobacterium-mediated transformation of tomatoes, the rate of tetraploid transgenic plants ranged from 24.5 to 80%, depending on both the genotype and the transformation procedure used (Ellul et al., 2003). In Arabidopsis, mutagenesis by T-DNA insertion can cause large-scale genomic rearrangements (Pucker et al., 2021). Therefore, this risk does not substantially undercut the value of protoplast regeneration and other transformation platforms as an excellent tool for gene editing.

CRISPR genome editing techniques can directly edit a target gene to create favorable traits in various crops, which opens the door to fast breeding of existing commercial varieties. However, most fruits, vegetables, and flowers are polyploid, heterozygous, asexually propagated, and/or have a long juvenile phase. CRISPR-mediated genome editing using protoplasts circumvents many of these problems and provides a material that is amenable to transgene-free products. Protoplasts provide a means to generate foreign-DNA-free mutants, which will improve their commercial value and avoid the difficult and time-consuming task of progeny hybridization. The major bottleneck with this technique is protoplast regeneration for various crops. On the other hand, there are currently no examples of protoplast fusion and application in gene-editing. This may also be considered to be one future direction for polyploidization, to create novel variety, and crop domestication. If these technical barriers can be overcome, CRISPR-mediated genome editing of protoplasts may usher in a new era of plant breeding.

## Data Availability

The original contributions presented in the study are included in the article/Supplementary Material, further inquiries can be directed to the corresponding author.

## References

[B1] Abdullah R. Cocking E. C. Thompson J. A. (1986). Efficient Plant Regeneration from rice Protoplasts through Somatic Embryogenesis. Nat. Biotechnol. 4, 1087–1090. 10.1038/nbt1286-1087

[B2] Abudayyeh O. O. Gootenberg J. S. Essletzbichler P. Han S. Joung J. Belanto J. J. (2017). RNA Targeting with CRISPR-Cas13. Nature 550, 280–284. 10.1038/nature24049 28976959 PMC5706658

[B3] Andersson M. Turesson H. Nicolia A. Fält A.-S. Samuelsson M. Hofvander P. (2017). Efficient Targeted Multiallelic Mutagenesis in Tetraploid Potato (*Solanum tuberosum*) by Transient CRISPR-Cas9 Expression in Protoplasts. Plant Cel. Rep. 36, 117–128. 10.1007/s00299-016-2062-3 PMC5206254 27699473

[B4] Andersson M. Turesson H. Olsson N. Fält A.-S. Ohlsson P. Gonzalez M. N. (2018). Genome Editing in Potato via CRISPR-Cas9 Ribonucleoprotein Delivery. Physiol. Plantarum 164, 378–384. 10.1111/ppl.12731 29572864

[B5] Bruznican S. Eeckhaut T. Van Huylenbroeck J. De Keyser E. De Keyser E. De Clercq H. (2021). An Asymmetric Protoplast Fusion and Screening Method for Generating Celeriac Cybrids. Sci. Rep. 11, 4553. 10.1038/s41598-021-83970-y 33633203 PMC7907277

[B6] Carlson P. S. Smith H. H. Dearing R. D. (1972). Parasexual Interspecific Plant Hybridization. Proc. Natl. Acad. Sci. 69, 2292–2294. 10.1073/pnas.69.8.2292 16592009 PMC426920

[B7] Čermák T. Curtin S. J. Gil-Humanes J. Cegan R. Kono T. J. Y. Konecna E. (2017). A Multipurpose Toolkit to Enable Advanced Genome Engineering in Plants. Plant Cell 29, 1196–1217. 10.1105/tpc.16.00922 28522548 PMC5502448

[B8] Chambers R. Höfler K. (1931). Micrurgical Studies on the Tonoplast of *Allium cepa* . Protoplasma 12, 338–355. 10.1007/BF01618743

[B9] Chen L. Li W. Katin-Grazzini L. Ding J. Gu X. Li Y. (2018). A Method for the Production and Expedient Screening of CRISPR/Cas9-mediated Non-transgenic Mutant Plants. Hortic. Res. 5, 13. 10.1038/s41438-018-0023-4 29531752 PMC5834642

[B10] Chung H.-H. Shi S.-K. Huang B. Chen J.-T. (2017). Enhanced Agronomic Traits and Medicinal Constituents of Autotetraploids in *Anoectochilus Formosanus* Hayata, a Top-Grade Medicinal Orchid. Molecules 22, 1907. 10.3390/molecules22111907 PMC6150389 29112129

[B11] Cocking E. C. (1960). A Method for the Isolation of Plant Protoplasts and Vacuoles. Nature 187, 962–963. 10.1038/187962a0

[B12] Cocking E. C. (1972). Plant Cell Protoplasts-Isolation and Development. Annu. Rev. Plant Physiol. 23, 29–50. 10.1146/annurev.pp.23.060172.000333

[B13] Constabel F. (1976). Somatic Hybridization in Higher Plants. In Vitro 12, 743–748. 10.1007/BF02835449 1021549

[B14] Davey M. R. Anthony P. Power J. B. Lowe K. C. (2005). Plant Protoplasts: Status and Biotechnological Perspectives. Biotechnol. Adv. 23, 131–171. 10.1016/j.biotechadv.2004.09.008 15694124

[B15] Davey M. R. Cocking E. C. Freeman J. Pearce N. Tudor I. (1980). Transformation of *Petunia* Protoplasts by Isolated *Agrobacterium* Plasmids. Plant Sci. Lett. 18, 307–313. 10.1016/0304-4211(80)90121-2

[B16] De Bruyn C. Ruttink T. Eeckhaut T. Jacobs T. De Keyser E. Goossens A. (2020). Establishment of CRISPR/Cas9 Genome Editing in Witloof (*Cichorium Intybus* Var. Foliosum). Front. Genome Ed. 2, 604876. 10.3389/fgeed.2020.604876 PMC8525355 34713228

[B17] Demirer G. S. Zhang H. Goh N. S. González-Grandío E. Landry M. P. (2019). Carbon Nanotube-Mediated DNA Delivery without Transgene Integration in Intact Plants. Nat. Protoc. 14, 2954–2971. 10.1038/s41596-019-0208-9 31534231 PMC10496593

[B18] Deng J. Cui H. Zhi D. Zhou C. Xia G. (2007). Analysis of Remote Asymmetric Somatic Hybrids between Common Wheat and *Arabidopsis thaliana* . Plant Cel. Rep. 26, 1233–1241. 10.1007/s00299-007-0345-4 17406873

[B19] Desprez B. Chupeau M.-C. Vermeulen A. s. Delbreil B. Chupeau Y. Bourgin J.-P. (1995). Regeneration and Characterization of Plants Produced from Mature Tobacco Pollen Protoplasts via Gametosomatic Hybridization. Plant Cel. Rep. 14, 204–209. 10.1007/BF00233634 24190296

[B20] Eeckhaut T. Lakshmanan P. S. Deryckere D. Van Bockstaele E. Van Huylenbroeck J. (2013). Progress in Plant Protoplast Research. Planta 238, 991–1003. 10.1007/s00425-013-1936-7 23955146

[B21] Ellison E. E. Nagalakshmi U. Gamo M. E. Huang P.-j. Dinesh-Kumar S. Voytas D. F. (2020). Multiplexed Heritable Gene Editing Using RNA Viruses and mobile Single Guide RNAs. Nat. Plants 6, 620–624. 10.1038/s41477-020-0670-y 32483329

[B22] Ellul P. Garcia-Sogo B. Pineda B. Ríos G. Roig L. Moreno V. (2003). The Ploidy Level of Transgenic Plants in *Agrobacterium*-Mediated Transformation of Tomato Cotyledons (*Lycopersicon esculentum* L.Mill.) Is Genotype and Procedure Dependent. Theor. Appl. Genet. 106, 231–238. 10.1007/s00122-002-0928-y 12582848

[B23] Fossi M. Amundson K. Kuppu S. Britt A. Comai L. (2019). Regeneration of *Solanum tuberosum* Plants from Protoplasts Induces Widespread Genome Instability. Plant Physiol. 180, 78–86. 10.1104/pp.18.00906 30792232 PMC6501065

[B24] Gerszberg A. Hnatuszko-Konka K. Kowalczyk T. (2015). *In Vitro* regeneration of Eight Cultivars of *Brassica oleracea* Var. Capitata. *In Vitro* Cell.Dev.Biol.-Plant 51, 80–87. 10.1007/s11627-014-9648-7 25774081 PMC4352192

[B25] González M. N. Massa G. A. Andersson M. Turesson H. Olsson N. Fält A.-S. (2020). Reduced Enzymatic browning in Potato Tubers by Specific Editing of a Polyphenol Oxidase Gene via Ribonucleoprotein Complexes Delivery of the CRISPR/Cas9 System. Front. Plant Sci. 10, 1649. 10.3389/fpls.2019.01649 31998338 PMC6962139

[B26] Guo W. W. Prasad D. Cheng Y. J. Serrano P. Deng X. X. Grosser J. W. (2004). Targeted Cybridization in Citrus: Transfer of Satsuma Cytoplasm to Seedy Cultivars for Potential Seedlessness. Plant Cel. Rep. 22, 752–758. 10.1007/s00299-003-0747-x 14730385

[B27] Hsu C.-T. Cheng Y.-J. Yuan Y.-H. Hung W.-F. Cheng Q.-W. Wu F.-H. (2019). Application of Cas12a and nCas9-Activation-Induced Cytidine Deaminase for Genome Editing and as a Non-sexual Strategy to Generate Homozygous/multiplex Edited Plants in the Allotetraploid Genome of Tobacco. Plant Mol. Biol. 101, 355–371. 10.1007/s11103-019-00907-w 31401729

[B28] Hsu C.-T. Lee W.-C. Cheng Y.-J. Yuan Y.-H. Wu F.-H. Lin C.-S. (2021a). Genome Editing and Protoplast Regeneration to Study Plant-Pathogen Interactions in the Model Plant *Nicotiana Benthamiana* . Front. Genome Ed. 2, 39. 10.3389/fgeed.2020.627803 PMC8525392 34713245

[B29] Hsu C. T. Yuan Y. H. Lin Y. C. Lin S. Cheng Q. W. Wu F. H. (2021b). Targeted Gene Editing in Plants Using CRISPR-Cas9, Single-Stranded DNA Oligonucleotides, and Protoplast Regeneration. bioRxiv 2021, 434087. 10.1101/2021.03.09.434087

[B30] Kao K. N. Constabel F. Michayluk M. R. Gamborg O. L. (1974). Plant Protoplast Fusion and Growth of Intergeneric Hybrid Cells. Planta 120, 215–227. 10.1007/BF00390290 24442697

[B32] Kiister E. (1909). Uber die Verschmelzung nackter Protoplasten. Ber. Dtsch. Bot. Ges. 27, 589–598. 10.1111/j.1438-8677.1909.tb06760.x

[B34] Kim H. Kim S.-T. Ryu J. Kang B.-C. Kim J.-S. Kim S.-G. (2017). CRISPR/Cpf1-mediated DNA-free Plant Genome Editing. Nat. Commun. 8, 14406. 10.1038/ncomms14406 28205546 PMC5316869

[B35] Kirti P. B. Mohapatra T. Khanna H. Prakash S. Chopra V. L. (1995). *Diplotaxis Catholica* + *Brassica Juncea* Somatic Hybrids: Molecular and Cytogenetic Characterization. Plant Cel. Rep. 14, 593–597. 10.1007/BF00231945 24185604

[B36] Kochevenko A. Ratushnyak Y. Kornyeyev D. Stasik O. Porublyova L. Kochubey S. (2000). Functional Cybrid Plants of Lycopersicon Peruvianum Var ‘Dentatum’ with Chloroplasts of Lycopersicon esculentum. Plant Cel Rep. 19, 588–597. 10.1007/s002990050778 30754822

[B37] Krens F. A. Molendijk L. Wullems G. J. Schilperoort R. A. (1982). *In Vitro* transformation of Plant Protoplasts with Ti-Plasmid DNA. Nature 296, 72–74. 10.1038/296072a0

[B38] Kumari P. Singh K. P. Kumar S. Yadava D. K. (2020). Development of a Yellow-Seeded Stable Allohexaploid *Brassica* through Inter-generic Somatic Hybridization with a High Degree of Fertility and Resistance to *Sclerotinia sclerotiorum* . Front. Plant Sci. 11, 575591. 10.3389/fpls.2020.575591 33329636 PMC7732669

[B39] Laiq U. R. Ahuja P. S. Banerjee S. (1994). Fertile Somatic Hybrid between Sexually Incompatible *Hyoscyamus Muticus* and *Hyoscyamus Albus* . Plant Cel. Rep. 13, 537–540. 10.1007/BF00232952 24194136

[B40] Lazzeri P. A. Brettschneider R. Lührs R. Lörz H. (1991). Stable Transformation of Barley via PEG-Induced Direct DNA Uptake into Protoplasts. Theoret. Appl. Genet. 81, 437–444. 10.1007/BF00219433 24221308

[B41] Lee L.-Y. Fang M.-J. Kuang L.-Y. Gelvin S. B. (2008). Vectors for Multi-Color Bimolecular Fluorescence Complementation to Investigate Protein-Protein Interactions in Living Plant Cells. Plant Methods 4, 24. 10.1186/1746-4811-4-24 18922163 PMC2572157

[B42] Li J.-F. Norville J. E. Aach J. McCormack M. Zhang D. Bush J. (2013). Multiplex and Homologous Recombination-Mediated Genome Editing in *Arabidopsis* and *Nicotiana Benthamiana* Using Guide RNA and Cas9. Nat. Biotechnol. 31, 688–691. 10.1038/nbt.2654 23929339 PMC4078740

[B43] Liang Z. Chen K. Zhang Y. Liu J. Yin K. Qiu J.-L. (2018). Genome Editing of Bread Wheat Using Biolistic Delivery of CRISPR/Cas9 *In Vitro* Transcripts or Ribonucleoproteins. Nat. Protoc. 13, 413–430. 10.1038/nprot.2017.145 29388938

[B44] Lin C.-S. Chang W.-C. (1998). Micropropagation of *Bambusa Edulis* through Nodal Explants of Field-Grown Culms and Flowering of Regenerated Plantlets. Plant Cel. Rep. 17, 617–620. 10.1007/s002990050453 30736514

[B45] Lin C.-S. Hsu C.-T. Yang L.-H. Lee L.-Y. Fu J.-Y. Cheng Q.-W. (2018). Application of Protoplast Technology to CRISPR/Cas9 Mutagenesis: from Single-Cell Mutation Detection to Mutant Plant Regeneration. Plant Biotechnol. J. 16, 1295–1310. 10.1111/pbi.12870 29230929 PMC5999315

[B46] Lin C.-S. Lin C.-C. Chang W.-C. (2004). Effect of Thidiazuron on Vegetative Tissue-Derived Somatic Embryogenesis and Flowering of Bamboo *Bambusa Edulis* . Plant Cell, Tissue Organ. Cult. 76, 75–82. 10.1023/A:1025848016557

[B47] Lin Q. Zong Y. Xue C. Wang S. Jin S. Zhu Z. (2020). Prime Genome Editing in rice and Wheat. Nat. Biotechnol. 38, 582–585. 10.1038/s41587-020-0455-x 32393904

[B48] Lin Y.-C. Li W. Chen H. Li Q. Sun Y.-H. Shi R. (2014). A Simple Improved-Throughput Xylem Protoplast System for Studying wood Formation. Nat. Protoc. 9, 2194–2205. 10.1038/nprot.2014.147 25144270

[B49] Ling J.-T. Iwamasa M. (1994). Somatic Hybridization between *Citrus Reticulata* and *Citropsis Gabunensis* through Electrofusion. Plant Cel. Rep. 13, 493–497. 10.1007/BF00232943 24194127

[B50] Louzada E. S. Grosser J. W. Gmitter F. G. Jr. (1993). Intergeneric Somatic Hybridization of Sexually Incompatible Parents: *Citrus Sinensis* and *Atalantia Ceylanica* . Plant Cel. Rep. 12, 687–690. 10.1007/BF00233420 24201965

[B51] Lowder L. G. Zhang D. Baltes N. J. Paul J. W. III. Tang X. Zheng X. (2015). A CRISPR/Cas9 Toolbox for Multiplexed Plant Genome Editing and Transcriptional Regulation. Plant Physiol. 169, 971–985. 10.1104/pp.15.00636 26297141 PMC4587453

[B52] Maher M. F. Nasti R. A. Vollbrecht M. Starker C. G. Clark M. D. Voytas D. F. (2020). Plant Gene Editing through De Novo Induction of Meristems. Nat. Biotechnol. 38, 84–89. 10.1038/s41587-019-0337-2 31844292 PMC6954279

[B53] Márton L. Wullems G. J. Molendijk L. Schilperoort R. A. (1979). *In Vitro* transformation of Cultured Cells from *Nicotiana Tabacum* by *Agrobacterium Tumefaciens* . Nature 277, 129–131. 10.1038/277129a0

[B54] Marx V. (2016). Plants: a Tool Box of Cell-Based Assays. Nat. Methods 13, 551–554. 10.1038/nmeth.3900 27355791

[B56] Nadakuduti S. S. Enciso-Rodríguez F. (2021). Advances in Genome Editing with CRISPR Systems and Transformation Technologies for Plant DNA Manipulation. Front. Plant Sci. 11, 637159. 10.3389/fpls.2020.637159 33519884 PMC7840963

[B57] Nagata T. Nemoto Y. Hasezawa S. (1992). Tobacco BY-2 Cell Line as the "HeLa" Cell in the Cell Biology of Higher Plants. Int. Rev. Cytol. 132, 1–30. 10.1016/S0074-7696(08)62452-3

[B58] Narasimhulu S. B. Kirti P. B. Prakash S. Chopra V. L. (1992). Resynthesis of *Brassica Carinata* by Protoplast Fusion and Recovery of a Novel Cytoplasmic Hybrid. Plant Cel. Rep. 11, 428–432. 10.1007/BF00234376 24201548

[B59] Nicolia A. Fält A.-S. Hofvander P. Andersson M. (2021). Protoplast-based Method for Genome Editing in Tetraploid Potato. Methods Mol. Biol. 2264, 177–186. 10.1007/978-1-0716-1201-9_12 33263910

[B60] Park S. C. Park S. Jeong Y. J. Lee S. B. Pyun J. W. Kim S. (2020). Simultaneous Targeting of Duplicated Genes in Petunia Protoplasts for Flower Color Modification via CRISPR-Cas9 Ribonucleoproteins. Plant Cel. Rep. 40 (6), 1037–1045. 10.1007/s00299-020-02593-1 32959126

[B61] Paszkowski J. Shillito R. D. Saul M. Mandák V. Hohn T. Hohn B. (1984). Direct Gene Transfer to Plants. EMBO J. 3, 2717–2722. 10.1002/j.1460-2075.1984.tb02201.x 16453573 PMC557758

[B62] Power J. B. Cummins S. E. Cocking E. C. (1970). Fusion of Isolated Plant Protoplasts. Nature 225, 1016–1018. 10.1038/2251016a0 16056900

[B63] Pucker B. Kleinbölting N. Weisshaar B. (2021). Large Scale Genomic Rearrangements in Selected *Arabidopsis thaliana* T-DNA Lines Are Caused by T-DNA Insertion Mutagenesis. bioRxiv 2021, 43375. 10.1101/2021.03.03.433755 PMC8348815 34362298

[B64] Roest S. Gilissen L. J. W. (1989). Plant Regeneration from Protoplasts: a Literature Review. Acta Botanica Neerlandica 38, 1–23. 10.1111/j.1438-8677.1989.tb01907.x

[B65] Ruesink A. W. (1971). The Plasma Membrane of *Avena* Coleoptile Protoplasts. Plant Physiol. 47, 192–195. 10.1104/pp.47.2.192 16657593 PMC365839

[B66] Shan Q. Wang Y. Li J. Zhang Y. Chen K. Liang Z. (2013). Targeted Genome Modification of Crop Plants Using a CRISPR-Cas System. Nat. Biotechnol. 31, 686–688. 10.1038/nbt.2650 23929338

[B67] Sheen J. (2001). Signal Transduction in maize and *Arabidopsis* Mesophyll Protoplasts. Plant Physiol. 127, 1466–1475. 10.1104/pp.01082010.1104/pp.127.4.1466 11743090 PMC1540179

[B68] Shillito R. D. Saul M. W. Paszkowski J. Müller M. Potrykus I. (1985). High Efficiency Direct Gene Transfer to Plants. Nat. Biotechnol. 3, 1099–1103. 10.1038/nbt1285-1099

[B69] Shimatani Z. Kashojiya S. Takayama M. Terada R. Arazoe T. Ishii H. (2017). Targeted Base Editing in rice and Tomato Using a CRISPR-Cas9 Cytidine Deaminase Fusion. Nat. Biotechnol. 35, 441–443. 10.1038/nbt.3833 28346401

[B70] Sigeno A. Hayashi S. Terachi T. Yamagishi H. (2009). Introduction of Transformed Chloroplasts from Tobacco into Petunia by Asymmetric Cell Fusion. Plant Cel. Rep. 28, 1633–1640. 10.1007/s00299-009-0763-6 19727738

[B71] Sretenovic S. Pan C. Tang X. Zhang Y. Qi Y. (2021). Rapid Vector Construction and Assessment of BE3 and Target-AID C to T Base Editing Systems in rice Protoplasts. Methods Mol. Biol. 2238, 95–113. 10.1007/978-1-0716-1068-8_7 33471327

[B72] Takahata Y. Takeda T. (1990). Intergeneric (Intersubtribe) Hybridization between *Moricandia Arvensis* and *Brassica* A and B Genome Species by Ovary Culture. Theoret. Appl. Genet. 80, 38–42. 10.1007/BF00224013 24220808

[B73] Takebe I. Labib G. Melchers G. (1971). Regeneration of Whole Plants from Isolated Mesophyll Protoplasts of Tobacco. Naturwissenschaften 58, 318–320. 10.1007/BF00624737

[B74] Tang X. Lowder L. G. Zhang T. Malzahn A. A. Zheng X. Voytas D. F. (2017). Correction: A CRISPR-Cpf1 System for Efficient Genome Editing and Transcriptional Repression in Plants. Nat. Plants 3, 17103. 10.1038/nplants.2017.103 28628131

[B75] Toda E. Koiso N. Takebayashi A. Ichikawa M. Kiba T. Osakabe K. (2019). An Efficient DNA- and Selectable-marker-free Genome-Editing System Using Zygotes in rice. Nat. Plants 5, 363–368. 10.1038/s41477-019-0386-z 30911123

[B76] Tuncel A. Corbin K. R. Ahn‐Jarvis J. Harris S. Hawkins E. Smedley M. A. (2019). Cas9‐mediated Mutagenesis of Potato Starch‐branching Enzymes Generates a Range of Tuber Starch Phenotypes. Plant Biotechnol. J. 17, 2259–2271. 10.1111/pbi.13137 31033104 PMC6835119

[B77] Varshney R. K. Nayak S. N. May G. D. Jackson S. A. (2009). Next-generation Sequencing Technologies and Their Implications for Crop Genetics and Breeding. Trends Biotechnol. 27, 522–530. 10.1016/j.tibtech.2009.05.006 19679362

[B78] Woo J. W. Kim J. Kwon S. I. Corvalán C. Cho S. W. Kim H. (2015). DNA-free Genome Editing in Plants with Preassembled CRISPR-Cas9 Ribonucleoproteins. Nat. Biotechnol. 33, 1162–1164. 10.1038/nbt.3389 26479191

[B79] Wu F.-H. Shen S.-C. Lee L.-Y. Lee S.-H. Chan M.-T. Lin C.-S. (2009). Tape-Arabidopsis Sandwich - a Simpler Arabidopsis Protoplast Isolation Method. Plant Methods 5, 16. 10.1186/1746-4811-5-16 19930690 PMC2794253

[B80] Wu J.-H. Ferguson A. R. Mooney P. A. (2005). Allotetraploid Hybrids Produced by Protoplast Fusion for Seedless Triploid Citrus Breeding. Euphytica 141, 229–235. 10.1007/s10681-005-7009-7

[B81] Wullems G. J. Molendijk L. Ooms G. Schilperoort R. A. (1981a). Differential Expression of crown Gall Tumor Markers in Transformants Obtained after *In Vitro Agrobacterium Tumefaciens*-induced Transformation of Cell wall Regenerating Protoplasts Derived from *Nicotiana Tabacum* . Proc. Natl. Acad. Sci. 78, 4344–4348. 10.1073/pnas.78.7.4344 16593059 PMC319786

[B82] Wullems G. J. Molendijk L. Ooms G. Schilperoort R. A. (1981b). Retention of Tumor Markers in F1 Progeny Plants from *In Vitro* Induced Octopine and Nopaline Tumor Tissues. Cell 24, 719–727. 10.1016/0092-8674(81)90098-2 7249079

[B83] Yoo S.-D. Cho Y.-H. Sheen J. (2007). *Arabidopsis* Mesophyll Protoplasts: a Versatile Cell System for Transient Gene Expression Analysis. Nat. Protoc. 2, 1565–1572. 10.1038/nprot.2007.199 17585298

[B84] Yu J. Tu L. Subburaj S. Bae S. Lee G.-J. (2020). Simultaneous Targeting of Duplicated Genes in *Petunia* Protoplasts for Flower Color Modification via CRISPR-Cas9 Ribonucleoproteins. Plant Cel. Rep. 40, 1037–1045. 10.1007/s00299-020-02593-1 32959126

[B85] Zhao X. Jayarathna S. Turesson H. Fält A.-S. Nestor G. González M. N. (2021). Amylose Starch with No Detectable Branching Developed through DNA-free CRISPR-Cas9 Mediated Mutagenesis of Two Starch Branching Enzymes in Potato. Sci. Rep. 11, 4311. 10.1038/s41598-021-83462-z 33619312 PMC7900246

[B86] Zhou A. Xia G. (2005). Introgression of the Haynaldia Villosa Genome into γ-ray-induced Asymmetric Somatic Hybrids of Wheat. Plant Cel. Rep. 24, 289–296. 10.1007/s00299-005-0922-3 15933881

[B87] Zhu H. Li C. Gao C. (2020). Applications of CRISPR-Cas in Agriculture and Plant Biotechnology. Nat. Rev. Mol. Cel. Biol. 21, 661–677. 10.1038/s41580-020-00288-9 32973356

[B88] Zimmermann U. Scheurich P. (1981). High Frequency Fusion of Plant Protoplasts by Electric fields. Planta 151, 26–32. 10.1007/BF00384233 24301666

